# Older Adults’ Strengths as Leaders of Age-Friendly Community Initiatives

**DOI:** 10.1093/geroni/igaf122.1694

**Published:** 2025-12-31

**Authors:** Emily Greenfield, Uri Amir-Koren, Natalie Pope

**Affiliations:** Rutgers University, New Brunswick, New Jersey, United States; Rutgers University, New Brunswick, New Jersey, United States; Rutgers University, New Brunswick, New Jersey, United States

## Abstract

The global age-friendly movement has long positioned older residents as central to age-friendly community (AFC) initiatives at the local level, yet there has been limited empirical exploration and theorizing into the ways older adults are positioned to advance this work in actual practice. To address this gap, our study aimed to understand the strengths older adults bring, from their own perspectives, to their age-friendly involvement. We explored this area using data from qualitative interviews with 23 older adults across four states in the United States. All study participants were in a leadership capacity for their community’s age-friendly initiative. Based on iterative rounds of reflexive thematic analysis, findings indicated how older adults leverage a variety of developmental strengths to advance age-friendly efforts at the local level, such as long-standing relationships with key community leaders and volunteers, personal experiences of challenges and opportunities for aging in the context of their communities, and past professional skills of relevance to advancing age-friendly goals and activities. We interpret these findings through transdisciplinary theoretical perspectives from social gerontology and beyond, such as socioemotional selectivity theory and continuity theory. We also discuss these findings in terms of their value in helping to identify and promote more optimal opportunities for older adults’ civic contributions through AFC initiatives, as well as how this research helps to address simultaneous attention to concerns about devolution and opportunities for empowerment as part of the AFC movement.

